# Studying the dynamics of interbeat interval time series of healthy and congestive heart failure subjects using scale based symbolic entropy analysis

**DOI:** 10.1371/journal.pone.0196823

**Published:** 2018-05-17

**Authors:** Imtiaz Awan, Wajid Aziz, Imran Hussain Shah, Nazneen Habib, Jalal S. Alowibdi, Sharjil Saeed, Malik Sajjad Ahmed Nadeem, Syed Ahsin Ali Shah

**Affiliations:** 1 Department of Computer Sciences & Information Technology, University of Azad Jammu & Kashmir, Muzaffarabad, Azad Kashmir, Pakistan; 2 Department of Computer Science, Faculty of Computing &IT, University of Jeddah, Jeddah, Saudi Arabia; 3 Department of Economics, University of Bath, Bath, United Kingdom; 4 Department of Sociology & Rural Development, University of Azad Jammu & Kashmir, Muzaffarabad, Azad Kashmir, Pakistan; Virginia Commonwealth University, UNITED STATES

## Abstract

Considerable interest has been devoted for developing a deeper understanding of the dynamics of healthy biological systems and how these dynamics are affected due to aging and disease. Entropy based complexity measures have widely been used for quantifying the dynamics of physical and biological systems. These techniques have provided valuable information leading to a fuller understanding of the dynamics of these systems and underlying stimuli that are responsible for anomalous behavior. The single scale based traditional entropy measures yielded contradictory results about the dynamics of real world time series data of healthy and pathological subjects. Recently the multiscale entropy (MSE) algorithm was introduced for precise description of the complexity of biological signals, which was used in numerous fields since its inception. The original MSE quantified the complexity of coarse-grained time series using sample entropy. The original MSE may be unreliable for short signals because the length of the coarse-grained time series decreases with increasing scaling factor *τ*, however, MSE works well for long signals. To overcome the drawback of original MSE, various variants of this method have been proposed for evaluating complexity efficiently. In this study, we have proposed multiscale normalized corrected Shannon entropy (MNCSE), in which instead of using sample entropy, symbolic entropy measure NCSE has been used as an entropy estimate. The results of the study are compared with traditional MSE. The effectiveness of the proposed approach is demonstrated using noise signals as well as interbeat interval signals from healthy and pathological subjects. The preliminary results of the study indicate that MNCSE values are more stable and reliable than original MSE values. The results show that MNCSE based features lead to higher classification accuracies in comparison with the MSE based features.

## Introduction

Time series analysis has significantly contributed to the understanding of complex systems and has been broadly adopted in scientific research and engineering application [1]. Numerous techniques have been developed for characterizing the complex behavior from observational data, e.g., chaotic time series analysis [2], fractal analysis [3], traditional complexity measures [4–6], multiscale entropy [7–9] and a panel of complex network analysis techniques [1]. The traditional complexity measures such as approximate entropy [5], sample entropy [6] and permutation entropy [10] are single scale-based and fail to account for the dynamics of real world time series data of complex biological systems which exhibit patterns of change on multiple time scales [7]. To address this issue, Costa et al. [7] introduced multiscale entropy (MSE) algorithm for precise description of the complexity of biological signals at multiple temporal scales. Since then, MSE has been used in various fields including biomedical signal processing [7–9, 11], financial time series [12] and electro-seismic time series data [13].

For computation of MSE, the first step is to construct coarse-grained time series at multiple temporal scales; the second step is to estimate the complexity of each coarse-grained time series using sample entropy [7–9]. Several drawbacks in the original MSE algorithm have been observed by the researchers [11, 14–18]. The sample entropy requires 10^m^ and 30^m^ data points for reliable estimation of complexity, where *m* is the embedding dimension [8]. The length of the coarse-grained time series decreases with the increase in time scale, which may result in reduced statistical reliability of sample entropy at large temporal scales and may induce undefined entropy values. The computation of sample entropy depends on the similarity criterion ‘r’, which is taken as a percentage of the standard deviation of the time series. The presence of observational and dynamical noise changes the standard deviation of the time series and hence the similarity criterion, which are likely to provide misleading information about the complexity of the system. Furthermore, in the original MSE, the similarity criterion is computed from time series data at time scale one and is kept constant for all the coarse-grained time series, which according to some researchers is another drawback of the original MSE algorithm [11, 15].

To address the aforementioned drawbacks in the original MSE algorithm, various variants of this approach have been proposed time to time by researchers in order to estimate complexity [11, 14–18]. In some studies, different coarse-graining procedures have been introduced to reduce the variance of entropy estimates [15, 16] and in some other studies, different entropy estimates instead of the sample entropy were used [14, 17–19]. Aziz and Arif [14] used permutation entropy as an entropy estimate for analyzing the complexity of interbeat interval time series data of healthy and diseased subjects. Ahmed and Mandic [19] introduced multiscale multivariate sample entropy (MMSE), a generalization of MSE algorithm to account for both within and cross channel dependencies of multivariate time series data. Azami et al. [20] introduced refined composite multiscale fuzzy entropy (RCMFE) based on standard deviation and mean for quantifying the dynamical properties of spread and mean, respectively, over multiple temporal scales. The results indicate that the RCMFE values are more stable and reliable than the original MSE values.

In this study, symbolic entropy measure, the multiscale normalized symbolic entropy measure (MNCSE) is proposed to quantify the complexity of the physiological systems. Like other traditional complexity measures such as approximate entropy [5], sample entropy [6] and permutation entropy [10], NCSE [21–23] being single scale-based fail to account for the dynamics of real world time series data of complex biological systems which exhibit patterns of change at multiple time scales [8]. For computing MNCSE, mean of data points was used as a criterion for constructing coarse-grained time series at various temporal scales. A nearly identical study appeared, form the methods point of view [24] during the review process, in which multivariate multiscale symbolic entropy is proposed. Three main differences including coarse graining procedure followed, data symbolization techniques used and application area exist between the two studies. The main focus of the study [24] is to address the multiscale and multichannel dependence inherent in the time series data, whereas our methodology accounts for the multiple time scale inherent on a single channel data. The coarse grained time series is generated using mean of multichannel time series data in [24], and in our proposed method coarse grained time series is generated by taking average of single channel time series data at a specific temporal scale. Secondly, the two methodologies using different data symbolization techniques. Thirdly, research reported in [24] is applied for discriminating control and neurodegenerative disease subjects, whereas, application area of our study is cardiac interbeat interval time series data.

In the present study, performance of the multiscale entropy metrics MSE and MNCSE was evaluated using simulated noise signals and interbeat interval time series data of healthy and pathological groups [25]. The analysis of variations in the interbeat interval called heart rate variability analysis (HRV) has attracted a great deal of attraction and is a valuable tool to extract information about the physiological state of the subject [7, 9, 14, 23, 26]. In 1996, standards in the assessment, interpretation and clinical use of HRV have been published by the Task force of the ESC/NASPE [26]. The HRV analysis has been used in various clinical settings, however, its practical use in adult medicine has been made in two scenarios i.e., reduced HRV 1) as marker of the risk stratification after acute myocardial infarction (MI) and 2) initial warning sign of diabetic neuropathy [26]. In this paper, we focus on interbeat interval time of NSR and CHF subjects to evaluate the performance of MNCSE and compared results with MSE. The MNCSE provided better classification between NSR and CHF subjects compared to MSE. The results also demonstrated that MNCSE efficiently characterized the changes with aging and disease severity.

## Materials and methods

### Data sets

The performance of scale based entropy measures MSE and MNCSE was evaluated using simulated noise signals and interbeat interval time series data of healthy and pathological subjects. The white Gaussian noise (WGN) and 1/f noise signals are widely used signals to evaluate the performance of MSE metrics [7, 9]. The samples of WGN are randomly drawn from a Gaussian distribution and are statistically not correlated. The 1/f noise signals are characterized by equal energy per octave and its power spectral density is inversely proportional to the signal frequency. For generation of 1/f noise, we start with uniformly distributed white noise and calculated its fast Fourier transform (FFT), then 1/f distribution was imposed on the power spectrum and the inverse FFT was calculated [7].

The time series data of healthy and pathological subjects used in this study was taken from Physionet, the research resource for complex physiological signals [26]. Physionet was established in 1999 to stimulate research in the field of biomedical science and to make novel investigations in the study of complex physiologic signals [27]. The interbeat interval (IBI) time series data of healthy subjects comprised of 72 subjects in normal sinus rhythm (NSR) and 44 patients with congestive heart failure (CHF). The data sets are available at https://physionet.org/physiobank/database/. The NSR is the regular complex rhythm of heart in which electrical impulses initiate in the sinoatrial (SA) node and are then transmitted through atrioventricular (AV) node to bundle of His, bundle branches and Purkinje fibers [28]. Out of 72 NSR subjects, 54 were taken from the RR-interval normal sinus rhythm database and 18 from the MIT BIH normal sinus rhythm database [25]. The group studied consists of 35 men and 37 women, aged 54.6±16.2 years (mean ± SD) and range 20–78 years. ECG data were sampled at 128 Hz.

The CHF is a pathophysiological condition in which the heart is unable to pump enough blood to meet the needs of the body [29]. The kidneys receive less blood and filter out lesser fluid which builds up in the lungs, around the eyes and sometimes in the legs causing congestion. The time series data of 44 CHF subjects was taken from two databases (29 from the RR interval congestive heart failure database and 15 from the MIT-BIH Bidmic congestive heart failure database) [25]. The CHF subjects comprise 29 men and 15 women aged 55.5±11.4 (mean±SD), range 22–78 years. Fifteen recordings were sampled at 250 Hz and 29 recordings were sampled at 128 Hz. According to the New York Heart Association (NYHA) functional classification system, CHF subjects can be classified into four groups [29]. For class I, there is no limitation of physical activity, whereas for class II there is a slight limitation in physical activity. In Class III subjects, the severity of the disease is moderate and there is marked limitation of physical activity. The Class IV CHF subjects are unable to carry out physical activity with comfort and belong to severe disease category. To study dynamical changes with disease severity, the CHF subjects were divided into two categories. The CHF subjects with lesser disease severity comprise of 12 subjects belonging to NYHA classes I and II and CHF subjects with high disease severity comprise 32 subjects belonging to NYHA class III and IV.

### Multiscale normalized corrected shannon entropy (MNCSE)

The procedure for computation of MNCSE involves the construction of coarse-grained time series at a specific time scale τ, the transformation of coarse-grained time series into symbol sequences, and finally quantification using NCSE [21–23]. Recently multivariate multiscale symbolic entropy [24] is introduced, which is identical to our proposed approach. However, the two approaches differ in terms of coarse graining procedure followed, data symbolization techniques used and application.

**Fig 1:**
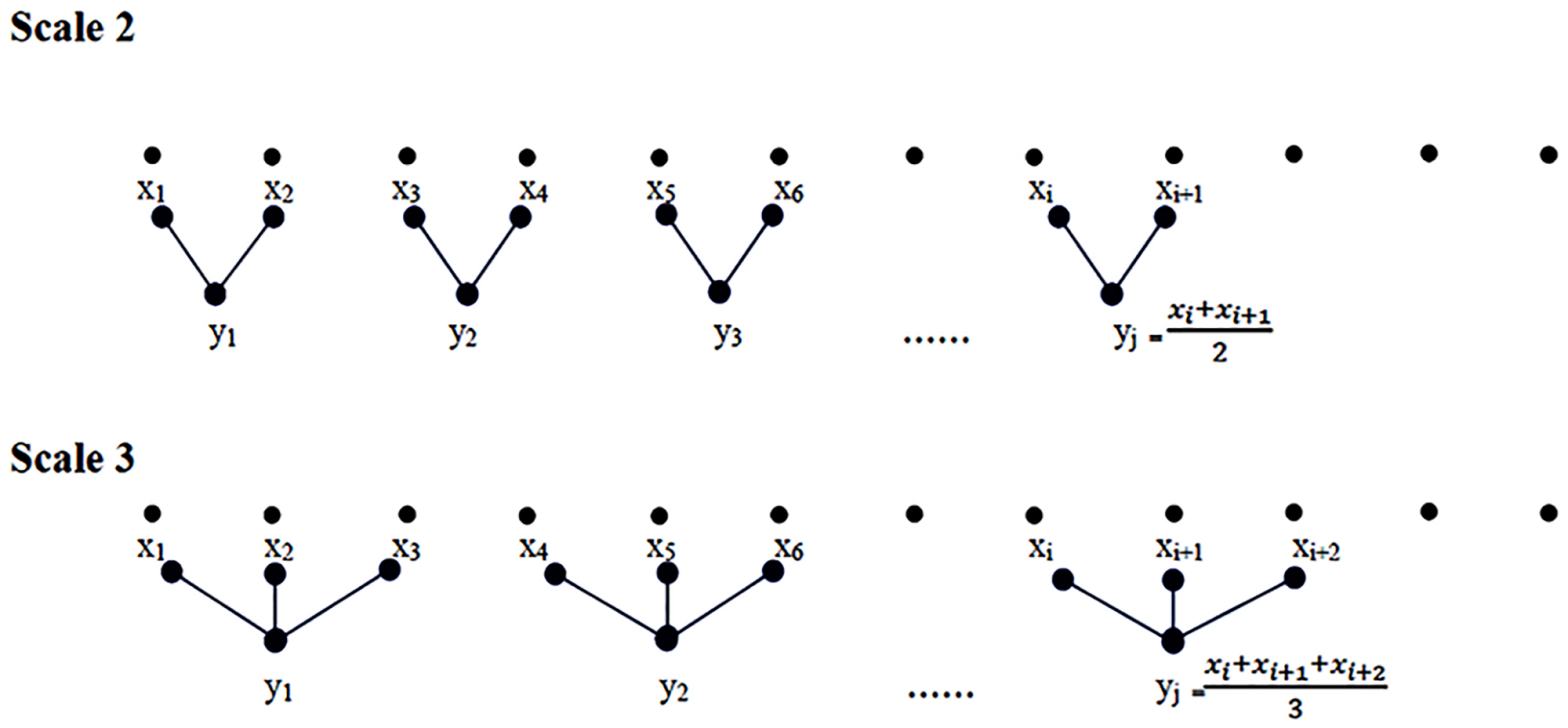
Illustration of coarse-graining procedure using mean of data points in a non-overlapping window equal in length to the time scale.

Given a time series *x* = {*x*_*i*_}, *i* = 1, …, *N*. The coarse-grained time series was constructed either by taking the average value of consecutive data points (Fig 1) in a non-overlapping window of data samples equal in length to the time scale.

The next step was to convert the coarse-grained time series into a symbol series for computation of MNCSE. The symbol sequences generated after transforming the time series *y* using quantization level *ξ* labelled from 0 to *ξ* − 1 are:

$$s(\xi ,\tau )={\{s}_{i}(\xi ,\tau )\;i=1,2,\ldots ,\;N-1\}$$ (1)

where *N* is the length of the coarse-grained time series at a scale factor τ. The time series was divided into two bins, using quantization level *ξ* = 2, and the value ‘1’ or ‘0’ was assigned to each bin according to the following criteria:

$$s_{i}\left(\xi ,\tau \right)=\{\begin{array}{rr} 1, & {\text{if}\;\text{y}}_{\text{i}}\geq \text{mean}\left(\text{y}\right)\\ 0, & \text{Otherwise} \end{array}$$ (2)

**Fig 2:**
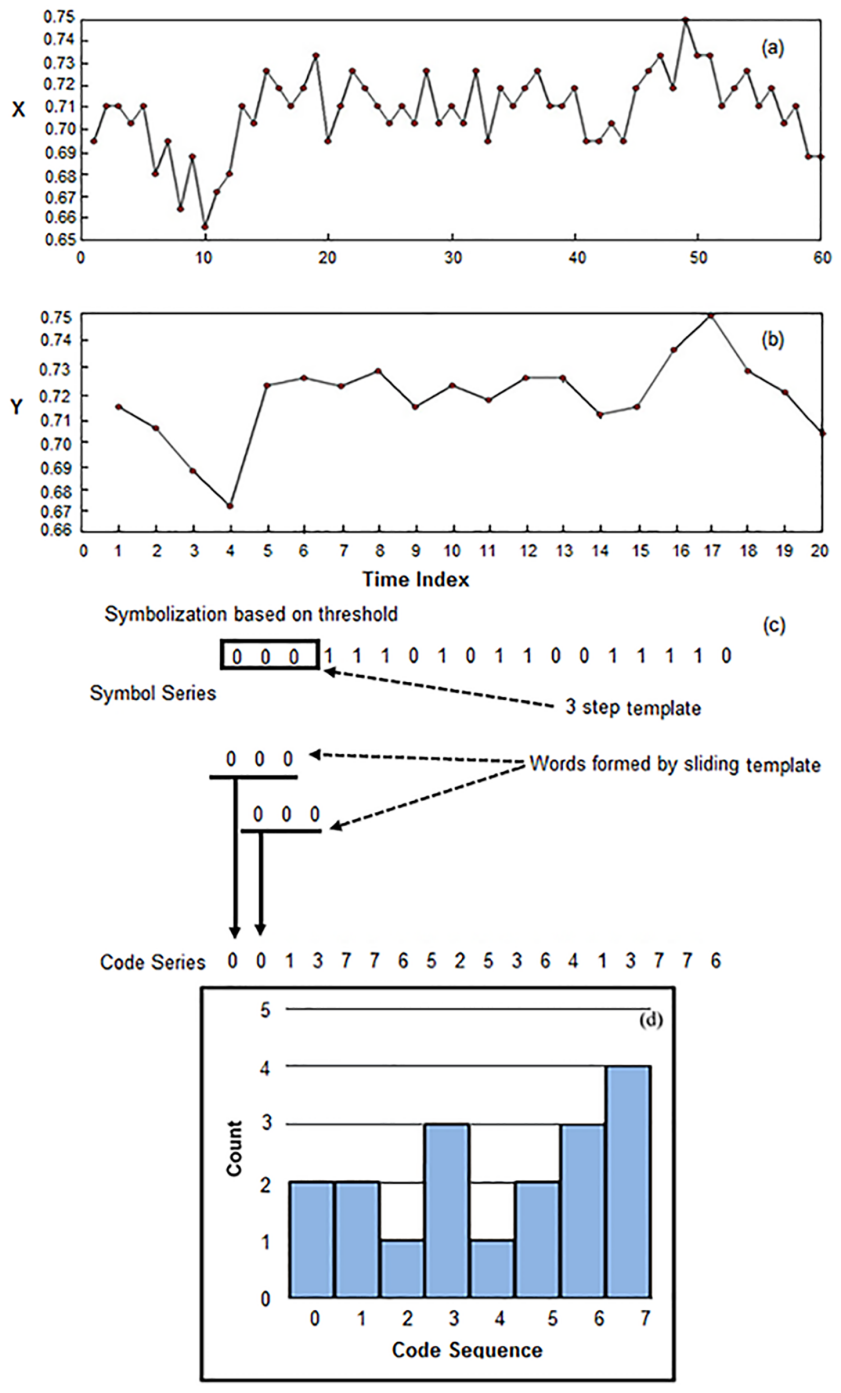
(a) Original times series (b) Coarse-grained time series (c) Data symbolization process (d) Histogram of symbol sequences.

The symbol sequences of words are constructed from any finite number of successive binary symbols of length *L*. Each possible sequence is represented by its binary number equivalent (or the decimal value) determined by the position of each symbol in the template of length L, using the relation:

$$w_{j}(L,\xi ,\tau )=\sum_{k=0}^{L-1}s_{k+j}(\xi ,\tau ).\xi^{k},\;j=1,2,\ldots ,n$$ (3)

where *n* is the length of the symbol time series. For quantization level ξ = 2 and word length *L* = 3, the number of all possible symbol sequence words is ξ^*L*^ = 2^3^ = 8 and the word range will be 0 to ξ^*L*^-1 (0 *to* 7). In Fig (2), the data symbolization process and histogram of symbol sequence words is illustrated for a time series coarse-grained at time scale 3, using quantization level ξ = 2 and word sequence length *L* = 3.

In the present study, the information theoretic measure normalized corrected Shannon entropy (NCSE) was used. The relation for computation Shannon entropy is:

$$SE(L,\xi ,\tau )=-\sum p_{w(L,\xi ,\tau )}\text{log}p_{w(L,\xi ,\tau )}$$ (4)

The estimate is affected by random error in numbers and also by a systematic error or bias. Eguia et al [30] reported the leading correction for the Shannon entropy and termed it Corrected Shannon Entropy (CSE).

$$CSE(L,\xi ,\tau )=SE(L,\xi ,\tau )+\frac{C_{R}-1}{2Mlog2}$$ (5)

where *M* is the total number of possible words and C_R_ is the number of occurring words among the possible words. The value of *CSE* is maximum for a certain word length *L* and quantization level ξ, when all *M* words occur with uniform distribution. Thus,

$${CSE}^{max}(L,\xi ,\tau )=-{\text{log}}_{2}\left(\frac{1}{M})+(\frac{M-1}{2Mlog2}\right)$$ (6)

Aziz and Arif [21] found that the maximum value of *CSE* will not be same for two different word lengths, because due to the increase in word length, the number of words *M* also increases, resulting in the increase in maximum value of *CSE*. Therefore, it is not possible to compare two values of *CSE* for two different word lengths at same threshold level and same quantization level. To overcome this problem, Normalized Corrected Shanon Entropy (NCSE) was proposed by Aziz and Arif [21–23]. The normalizing factor in NCSE is the CSE^max^(*L*,), which is the maximum value of *CSE* for a certain word length *L* and quantization level *ε*. The NCSE at multiple time scales is called MNCSE, and is determined as follows:

$$MNCSE(L,\xi ,\tau )=\frac{CSE(L,\xi ,\tau )}{{CSE}^{max}(L,\xi ,\tau )}$$ (7)

The value of *NCSE* will be in the range from 0 to 1 for any word length *L* and quantization level ξ. In Fig 2, the data symbolization and symbol sequence formation procedure using quantization level 2 and word length is illustrated in detail.

### Statistical analysis

The Kruskal-Wallis test, which is a non-parametric analogue of the Analysis of Variance (ANOVA) was used to find whether the differences between the medians of three or more categorical variables is statistically significant, using a p-value ≤0.05. For paired comparison, after the Kruskal-Wallis test we conducted a series of Wilcoxon Mann-Whitney tests to investigate which groups significantly differ. The area under receiver operating characteristic (AUC) curve was used to assess the degree of separation between various groups [31]. The AUC can take any value between 0 and 1; the closer the AUC value to 1, better the degree of separation between the groups. The practical lower limit of AUC is 0.5 at which the ROC curve will fall to the diagonal and the overall performance of the diagnostic test will rely on pure chance [31].

### Features selection and classification methods

In machine learning, classification is the process of identifying suitable class label (category) of a new observation on the basis of a model build using training set of data containing observations whose category-membership are known. During last few decades various machine learning techniques have been proposed for developing decision support systems for accurate classification of medical data. In machine learning literature 10 times 10-fold cross validation (10 by 10 FCV) and Leave-One-Out cross validation (LOOCV) are the common resampling techniques which are used when data set for analysis is small [32–34]. Therefore, in this study classification has been done in both of the above mentioned settings. The results of MNCSE & MSE at scale 1 & optimal scale have been used as features for the classification of healthy, elderly and diseased subjects. Support Vector Machine (SVM) with Radial Kernel [35], Random Forests (RF) [36] and k-Nearest Neighbour (kNN) [37] (with k = 3 because we have small sample size) algorithms have been used to build classification models. The presented results obtained by using 10 by 10 FCV, LOOCV and learning algorithms mentioned above demonstrated that feature values obtained from MNSCE gave more accurate separation of healthy, pathological and elderly groups as compared to those values obtained from MSE.

## Results

### Simulated noise signals

**Fig 3:**
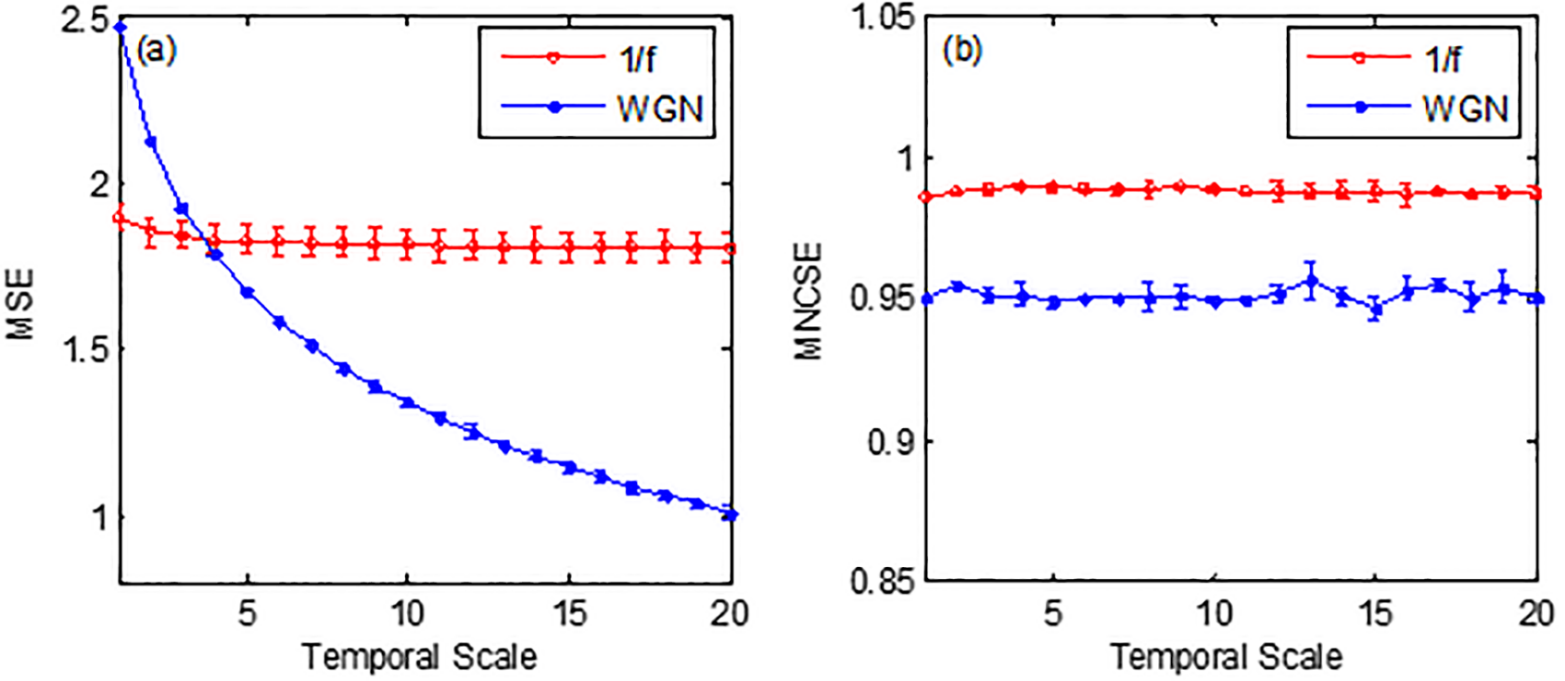
Mean ± SD values of the (a) MSE and (b) MNCSE computed from40 different WGN and 1/f noise signals.

The results of scale based entropy metrics MSE and MNCSE are depicted in Fig 3. The Symbols represent mean values of entropy for the 40 WGN and 1/f noise signals and error bars represent the standard deviation (SD).

It is evident from the Fig 3, MSE decreased monotonically with scale factor for WGN and remain constant for 1/f noise. The MSE values were higher for WGN at small temporal scales < 4 and as compared to 1/f noise values. The MNCSE values at all temporal scales were higher for 1/f noise than corresponding WGN values depicting that 1/f noise has more complex structures than WGN due to presence of long range correlations even at temporal scale 1.

### Sensitivity of MSE and MNCE with signal length

**Fig 4:**
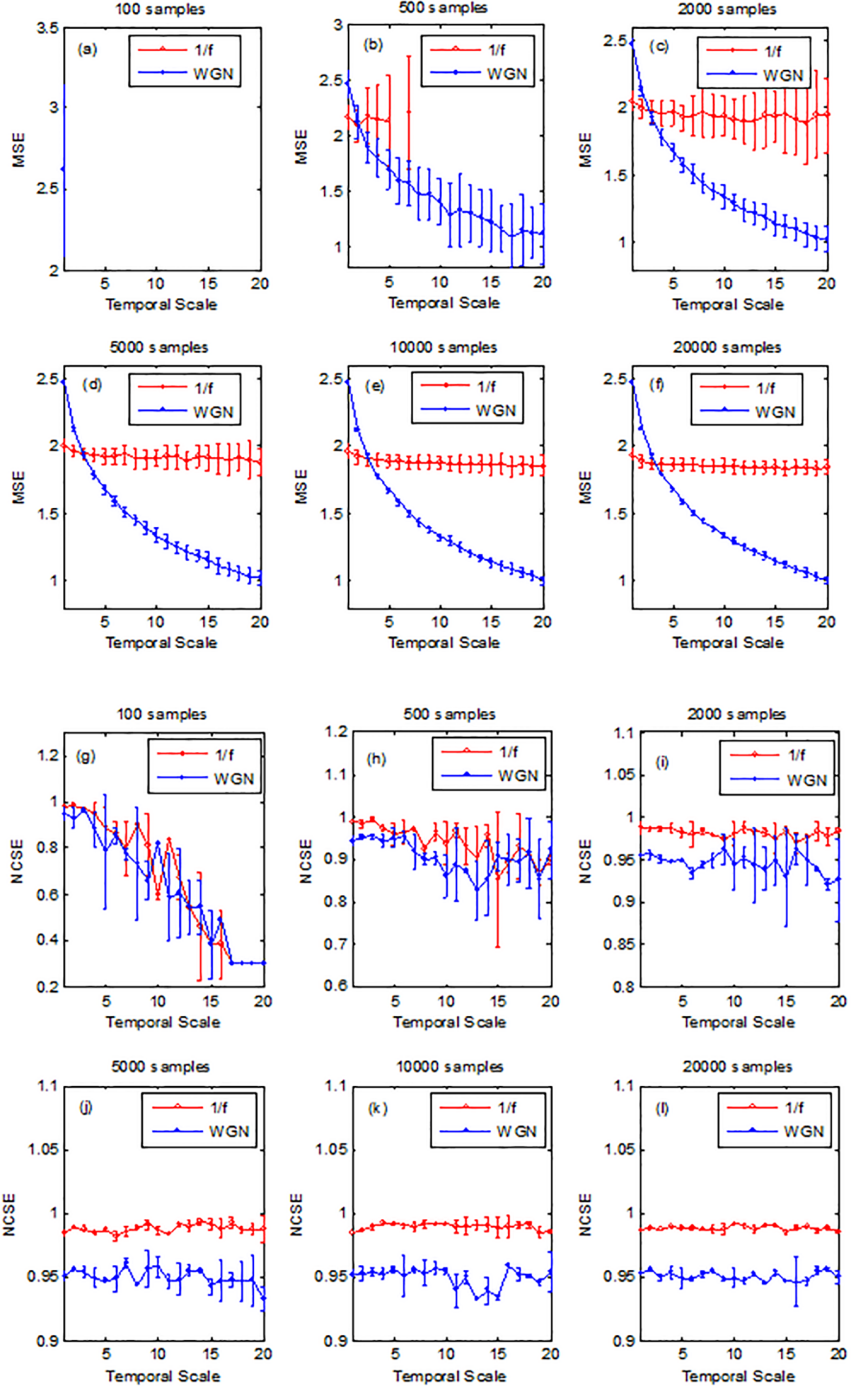
MSE (a-f) and MNCSE (g-l) as a function of data length, computed from 40 realizations of WGN and 1/f noise signals.

The sensitivity of MSE and MNCSE with signal length was evaluated using WGN and 1/f noise signals. Fig 4(a)–4(f) and 4(g)–4(l) respectively depict the MSE and MNCSE values for signal length 100, 500, 2000, 5000, 10000 and 20000 samples computed from 40 different realizations of WGN and 1/f noise signals. It is evident from Fig 4, for short duration signals such as 100 and 500 samples, MSE induced undefined entropy values, whereas MNSCE did not induce undefined entropy values for any of the signals lengths. An overlap of MNCSE values was found for short duration time series data of WGN and 1/f noise, however, for time series data ≥1000 samples, MNCSE was able to distinguish between these two signals at all the temporal scales. It is also evidences that MNCSE is computationally more efficient as compared to MSE.

### Interbeat interval time series data

In Table 1, mean ranks of MNCSE and MSE and their corresponding p-values along with area under ROC for NSR young, CHF class III-IV and AF subjects are presented at temporal scales 1 to 15. At scale 1, MNCSE corresponds to NCSE and MSE corresponds to sample entropy. Higher mean ranks correspond to higher entropy estimates and hence higher complexity. Both scales based on the sample and symbolic entropy measures were able to discriminate NSR young from other groups more significantly at a wide range of scales. At scale 1, the mean rank of NCSE values for NSR young subjects was smaller compared to mean ranks of CHF class III-IV, providing dynamically incorrect information. At temporal scales 2 and above, mean ranks of NSR young subjects was significantly higher than CHF class III-IV. The maximum separation between NSR subjects and CHF class III-IV was obtained at temporal scale 4 for both scale based measures. The level of significance and the area under the ROC curve for separating NSR young and CHF class III-IV subjects using MNCSE was 2.72×10^−06^ and 0.86 respectively, compared to 1.20×10^−05^ and 0.82 using MSE.

**Table 1:** Mean ranks, corresponding p-values and area under ROC curve for comparison of MNCSE and MSE at temporal scales 1 to 15 for quantifying the dynamics of NSR, CHF and young and elderly subjects.

**NSR Vs CHF**								
**Scale**	**MNCSE**				**MSE**			
	**NSR Young**	**CHF**	**p-value**	**Area under ROC**	**NSR Young**	**CHF**	**p-value**	**AUC**
1	28.42	30.38	6.62×10^−01^	0.53	36.50	23.81	4.43×10^−03^	0.69
2	37.04	23.38	2.18×10^−03^	0.74	39.08	21.72	9.89×10^−05^	0.78
3	39.35	21.50	6.26×10^−05^	0.81	39.73	21.19	3.20×10^−05^	0.81
4	41.04	20.13	2.72×10^−06^	0.86	40.27	20.75	1.20×10^−05^	0.82
5	41.04	20.13	2.72×10^−06^	0.86	39.85	21.09	2.60×10^−05^	0.81
6	41.00	20.16	2.94×10^−06^	0.86	39.88	21.06	2.43×10^−05^	0.80
7	40.42	20.63	8.98×10^−06^	0.84	39.42	21.44	5.49×10^−05^	0.80
8	40.31	20.72	1.12×10^−05^	0.84	39.58	21.31	4.20×10^−05^	0.80
9	39.96	21.00	2.11×10^−05^	0.83	39.46	21.41	5.13×10^−05^	0.79
10	39.46	21.41	5.13×10^−05^	0.81	39.35	21.50	6.26×10^−05^	0.79
11	39.50	21.38	4.80×10^−05^	0.81	38.73	22.00	1.75×10^−05^	0.77
12	39.42	21.44	5.49×10^−05^	0.81	38.73	22.00	1.75×10^−05^	0.77
13	39.54	21.34	4.49×10^−05^	0.81	38.73	22.00	1.75×10^−05^	0.77
14	39.04	21.75	1.06×10^−05^	0.80	38.42	22.25	2.86×10^−05^	0.76
15	39.08	21.72	9.89×10^−05^	0.80	38.69	22.03	1.86×10^−05^	0.77
**NSR Young Vs NSR Old**								
**Scale**	**NSR Young**	**NSR Old**	**p-value**	**Area under ROC**	**NSR-Young**	**NSR Old**	**p-value**	**Area under ROC**
1	51.46	28.04	5.10×10^−06^	0.82	47.88	30.07	5.20×10^−04^	0.71
2	55.96	25.50	2.99×10^−09^	0.92	50.31	28.70	2.57×10^−05^	0.77
3	56.88	24.98	5.18×10^−10^	0.94	50.62	28.52	1.69×10^−05^	0.77
4	56.54	25.17	1.01×10^−09^	0.94	50.50	28.59	1.98×10^−05^	0.77
5	55.81	25.59	3.97×10^−09^	0.92	49.96	28.89	4.07×10^−05^	0.76
6	55.62	25.70	5.65×10^−09^	0.92	50.04	28.85	3.68×10^−05^	0.76
7	55.15	25.96	1.30×10^−08^	0.91	50.38	28.65	2.31×10^−05^	0.77
8	54.62	26.26	3.35×10^−08^	0.89	50.23	28.74	2.85×10^−05^	0.77
9	54.69	26.22	2.93×10^−08^	0.90	50.73	28.46	1.44×10^−05^	0.78
10	54.50	26.33	4.10×10^−08^	0.89	51.31	28.13	6.37×10^−05^	0.79
11	54.42	26.37	4.68×10^−08^	0.89	50.15	28.78	3.16×10^−05^	0.77
12	54.54	26.30	3.83×10^−08^	0.89	51.12	28.24	8.39×10^−06^	0.79
13	54.69	26.22	2.93×10^−08^	0.90	51.15	28.22	7.94×10^−06^	0.79
14	54.62	26.26	3.35×10^−08^	0.89	51.12	28.24	8.39×10^−06^	0.79
15	54.54	26.30	3.83×10^−08^	0.89	50.77	28.43	1.36×10^−05^	0.78

On comparing NSR young subjects with NSR elderly subjects, both scale-based measures provide dynamically more correct information at all temporal scales. The maximum separation between NSR elderly and young subjects was obtained at temporal scale 3, mean ranks (56.88 for young and 24.98 for elderly subjects), p-values5.18×10^−10^ and the AUC 0.94 using MNCSE. On the other hand, maximum separation between NSR elderly and young subjects is obtained at temporal scale 13, mean ranks (51.15 for young and 28.22 for elderly subjects), p-values 7.94×10^−06^ and AUC 0.79 using MNCSE.

**Fig 5:**
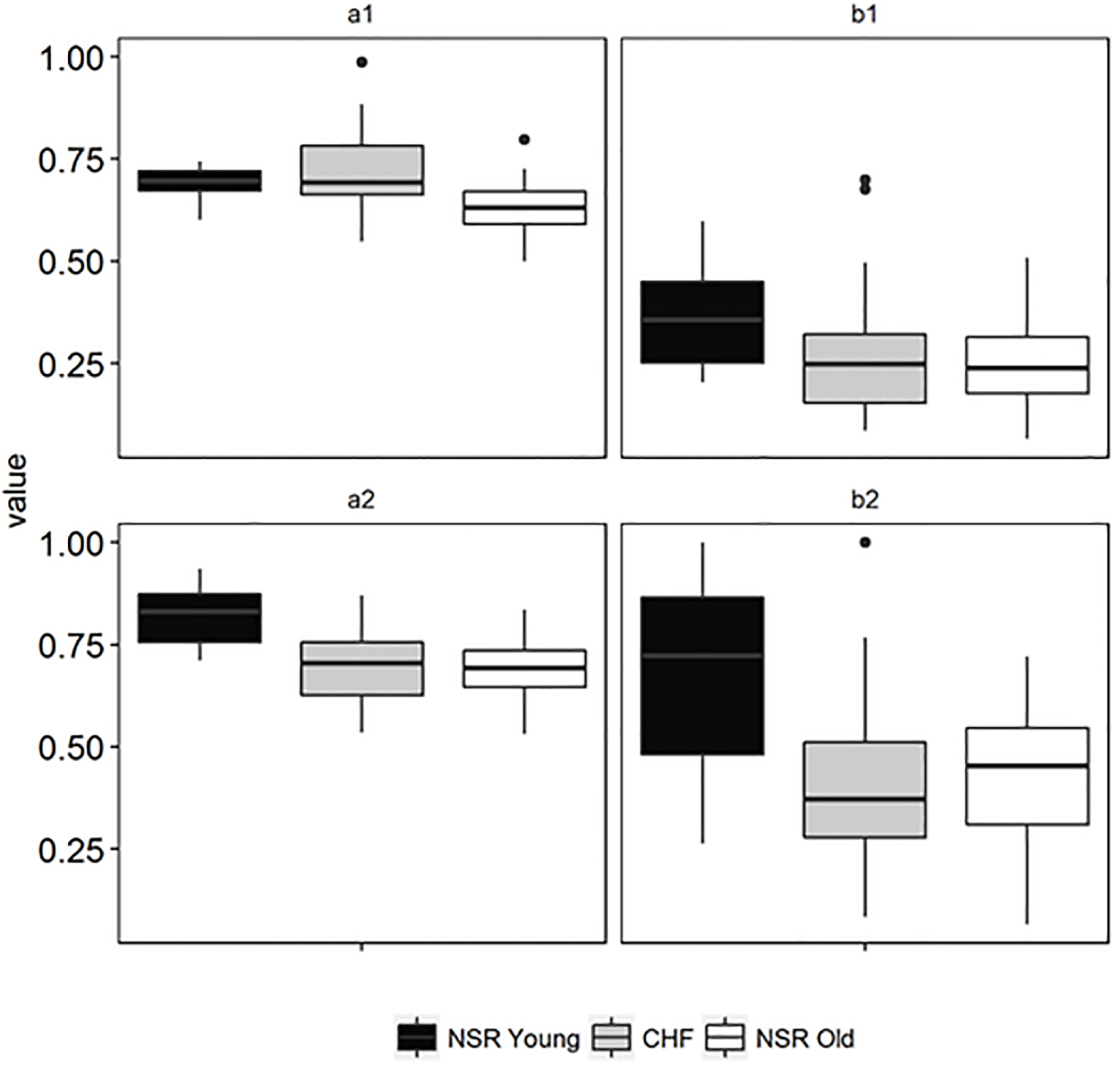
Mean ranks for comparison of a) MNCSE and b) MSE at temporal scales 1 to 15 for quantifying the dynamics of pathological with disease severity.

In Fig 5, the pattern of response for MNCSE (a1 and a2) and MSE (b1 and b2) at temporal scale 1 and at optimal temporal scale for differentiating healthy and stressed groups (CHF and elderly subjects) are shown using boxplot. Inside each box, the middle line represents the median value, while the upper and lower whiskers represent entropy estimate values outside the middle 50% and entropy estimates outside upper and lower whiskers represented by a dot (●) symbol are outliers. At temporal scale 1, the overlap of both entropy estimates was large, and at optimal temporal scale the overlap of entropy estimates has decreased. The decrease in the overlap of entropy estimate values suggests a difference between the healthy and stressed groups. The boxplots of MNCSE are comparatively shorter than the boxplot of MSE, depicting high level of agreement with each other within a group. Furthermore, the upper and lower whisker lengths of MNCSE boxplots are shorter than MSE boxplots, suggesting that variance of MNCSE is smaller. The smaller variance of MNCSE leads to greater reliability of this entropy estimate compared to MSE.

**Fig 6:**
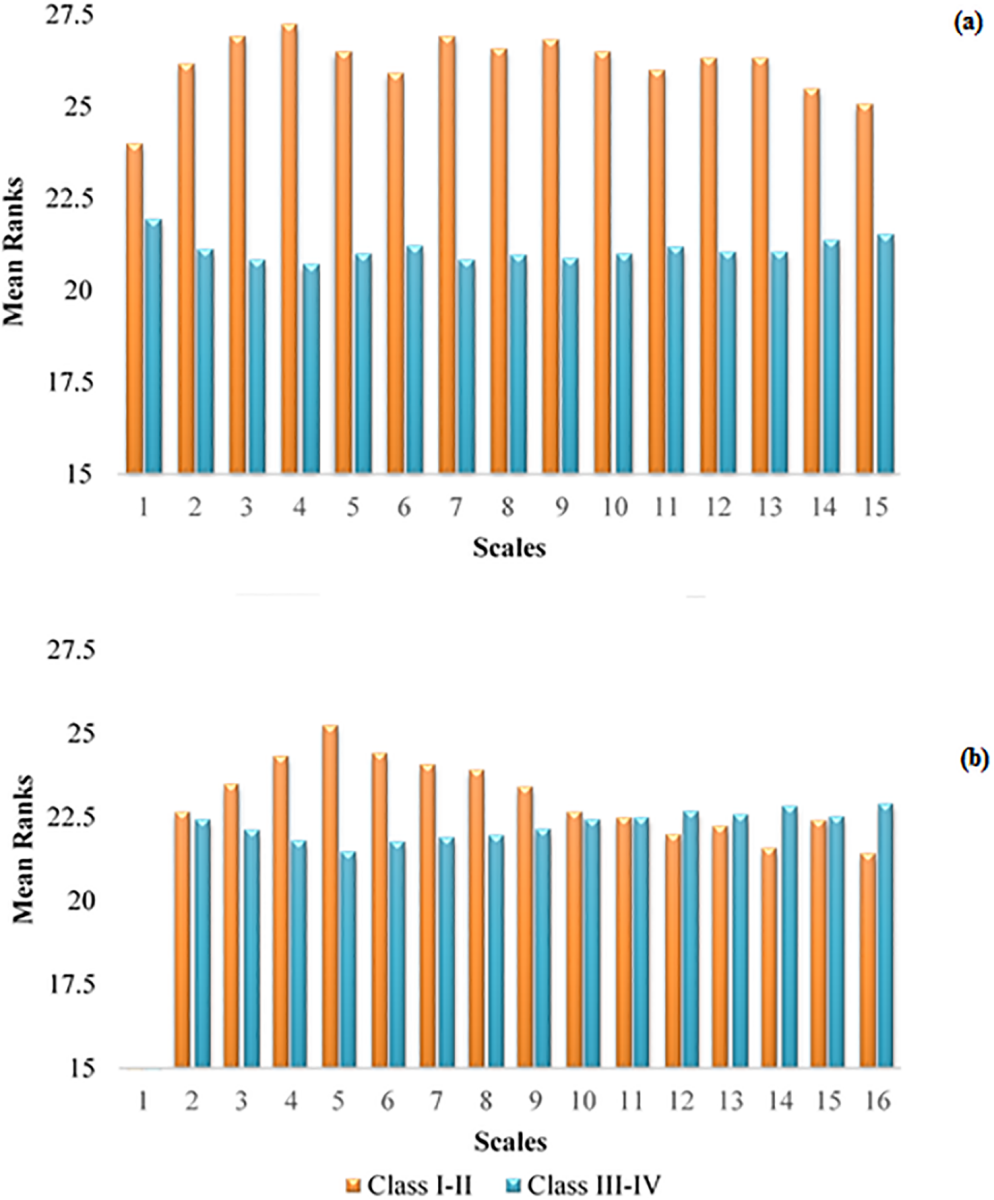
Mean ranks for comparison of a) MNCSE and b) MSE at temporal scales 1 to 15 for quantifying the dynamics of pathological with disease severity.

In Fig 6, a comparison of MNCSE and MSE is presented in order to quantify the dynamical changes of CHF subjects with disease severity. It is evident from the Fig 6 that the differences between both entropy estimates were smaller at scale 1, compared to entropy estimates at multiple time scale. The MNCSE provided was more robust in distinguishing CHF subjects of class I-II and CHF subjects class III-IV.

To investigate the predictability of healthy and diseased subjects on the basis of features extracted (mean values) from interbeat interval time series using MNCSE & MSE at scale 1 & optimal scale we used two setting namely 10 times 10-fold cross validation (10 by 10 FCV) and Leave-One-Out cross validation (LOOCV). Both 10 by 10 FCV and LOOCV are used by researchers to explore the performance of predictive models built on the basis of features extracted from time series data [30]. In each of these setting classification accuracy (CA) is used as a measure of a classifier’s performance. According to the notation of confusion matrix, CA was computed using the relation:

CA=[(TP+TN)/(TP+TN+FP+FN)]×100 (8)

Here TP is the count of correctly classified healthy subject, TN is of correctly classified diseased subjects, FP represents count of falsely classified healthy subject and FN is the count of falsely classified diseased subjects.

We used Support Vector Machine (SVM with Radial Kernel), Random Forests (RF) and *k*-Nearest Neighbour (*k*NN with *k* = 3 because we have small sample size) as learning algorithms to build predictive models for two scenarios (NSR Young vs CHF and NSR Young vs NSR Old) for each of 10 by 10 FCV and LOOCV settings. Here we discuss the results in two sets of experiments.

#### Set 01

**Fig 7:**
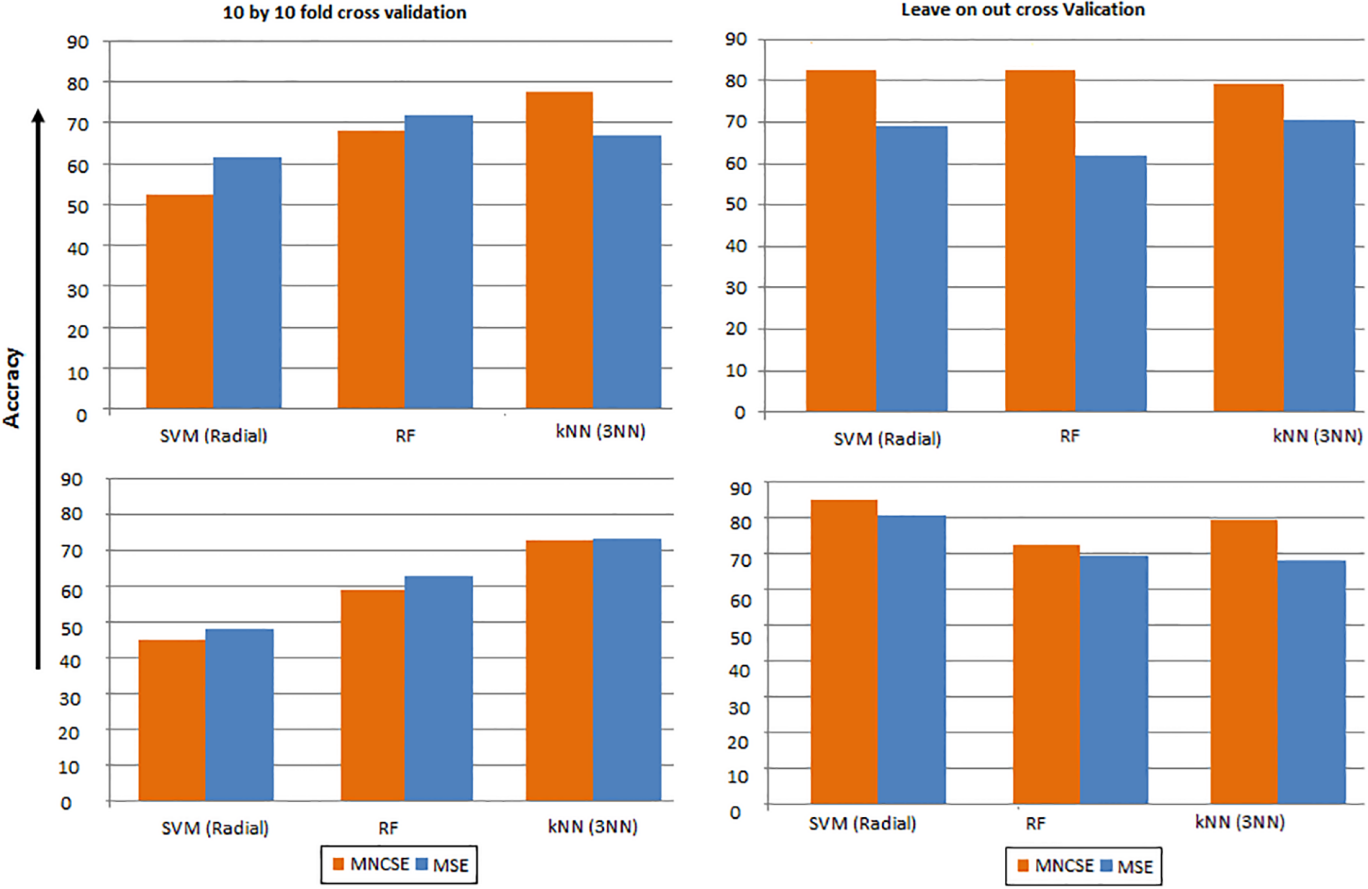
Classification accuracy (CA), computed using (a)10 by 10 FCV for separating NSR and CHF subjects (b) 10 by 10 FCV for separating NSR young and NSR elderly subjects (c) leave-one-out cross validation for separating NSR and CHF subjects (d) leave-one-out cross validation for separating NSR young and NSR elderly subjects.

Classically, in 10 FCV setting the available group of samples is randomly partitioned into 10 equal portions; nine of which are used for building predictive model and one for testing purposes. The procedure is repeated 10 times so each portion is used once for validation. In this way class labels for all samples in the group are obtained. However, this method may introduce bias due to random partition of the datasets. Therefore, to reduce bias in the cross-validation, we apply 10 FCV 10 times independently and take average to estimate final CA (Eq 8) for each of the scenarios mentioned above. Results of the simulations for 10 by 10 FCV are presented in Fig 7(a) and 7(b). Results show that for MNCSE maximum average CA (77.38%) is obtained using *k*NN based classifier and for NSR Young vs CHF group. Moreover, *k*NN based classifier is more accurate as compared to other two classifiers used in this analysis. For MSE maximum average CA is 71.98%. This accuracy is obtained from RF classifier and for the same NSR Young vs CHF group. These results reflects that features extracted using MNCSE from interbeat interval time series are more valuable for building predictive model to classify healthy and diseased subjects as compared to those extracted by MSE.

#### Set 02

In LOOCV one of the samples is used to test the performance of the predictive model while rest of the samples are used to construct the predictive model. This procedure is repeated for all the samples in the group and we have only one sample for performance evaluation each time. In this way the performance of the predictive models are measured for all the samples in the group. Results of LOOCV setting are shown in Fig 7(c) and 7(d). For features extracted by MNCSE the maximum computed average classification accuracy CA = 82.76% is again for NSR Young vs CHF scenario but using SVM and RF based classifiers. Results presented also show that SVM also give maximum average accuracy (84.72%) using features extracted by MSE technique. In the analysis of NSR Young vs CHF group, the classification models using LOOCV and MNCSE have extracted features, it is evident that predictive accuracies with two classifiers (SVM & RF) are better than those models built using LOOCV and features extracted using MSE. While comparing predictive accuracies of classification models for NSR Young vs NSR Old group, the results show that models build by using LOOCV and MNCSE based extracted features give better accuracy as compared to those build using LOOCV and MSE based extracted features. Here SVM (Radial) learning algorithm better learn than other two learning algorithms used in this work. The present results show that MNCSE is better technique for extracting features from interbeat interval time series data.

**Fig 8:**
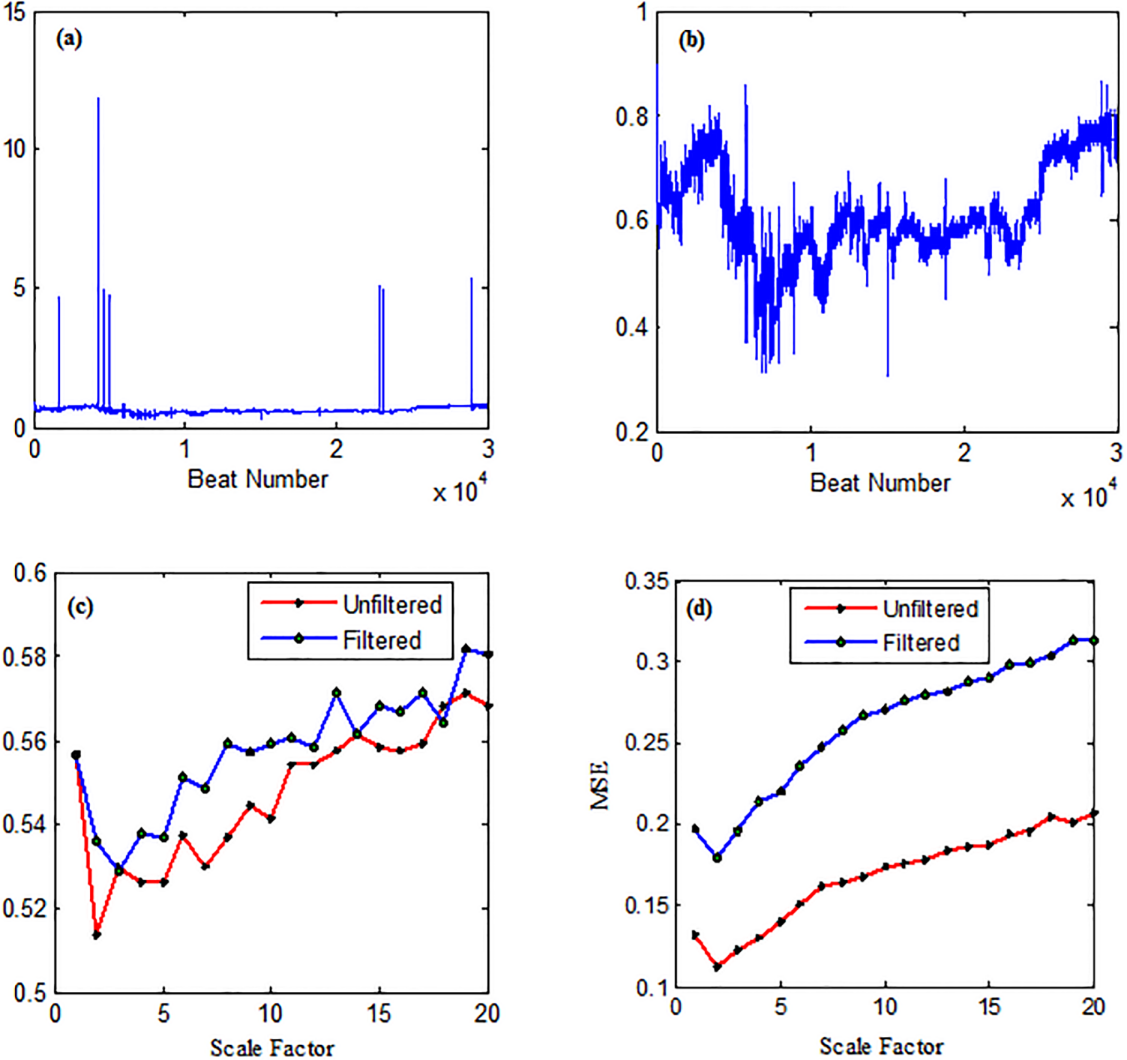
(a) RR interval time series from healthy subject (b) Time series obtained by excluding artifacts greater than 2s. (c) MNCSE analysis (d) MSE analysis.

The robustness of MNCSE was evaluated in the presence of artifacts and results were compared with MSE at various temporal scales (Fig 8). In Fig 8(a) first 30,000 interbeat interval time series of healthy subjects is shown. Time series shown in Fig 8(b) is obtained by excluding RR intervals greater than 2s. MSE is plotted as a function of scale factor for filtered and unfiltered time series is shown in Fig 8(c) and NCSE for filtered and unfiltered time series is shown in the Fig 8(d). It is clear from the Fig 8(c), that separation between filtered and unfiltered time series is for MSE at all the temporal scales, however, as shown in Fig 8(d), the difference between MNCSE values is very small. The results yielded that MNCSE is more robust than MSE in case of dynamical or observational noise.

## Discussion

The complexity analysis has widely been used to extract valuable hidden information from biological signals about the dynamics of these systems in health and disease [35, 8]. In healthy biological systems, the long evolutionary process increases the adaptive capability against most external perturbations in the dynamic environment reflecting complex dynamics [34]. The pathogenesis delimits the capability of the biological system to evolve with time, which results in the reduced adaptability reflecting loss of dynamical complexity [35, 8, 10]. Due to the hierarchy of structural sub-systems (numerous agents) and their coupling function, the biological systems exhibit complex patterns at multiple spatial and temporal scales [35, 8]. Thus the traditional single scale complexity measures [1, 2] may provide misleading results about the dynamic real world time series data. Costa [8] introduced multiscale entropy (MSE) and applied it to analyze cardiac rhythms [8, 10], gait dynamics [9] and analysis of coding DNA sequences [10]. The original MSE used sample entropy [2] as an entropy estimate, which suffered from numerous limitations. Various variants of MSE have been proposed by researchers for estimating complexity [4, 7, 11–13] either by using different coarse-graining procedures or by using different entropy estimates.

In this study, we have used the symbolic entropy measure NCSE as an entropy estimate instead of sample entropy at different temporal scales to quantify the complexity of simulated noise signals and interbeat interval time series data. The dynamics 1/f noise are more complex than WGN due to presence of long range correlation. The MNCSE values at all temporal scales were higher for 1/f noise than corresponding WGN values even at temporal scale 1. The results manifested that MNCSE showed higher complexity of 1/f noise signal at all temporal scales. The sensitivity of MSE and MNCSE with signal length was also evaluated using WGN and 1/f noise signals. The findings indicated that MSE induced undefined entropy for short length time series data, especially at large temporal scales. On the other hand, MNCSE handled this issue and did not induce undefined estimates. The coarse-graining procedure down sample the averaged data and hence length of coarse grained timed series by a scale factor τ. For reliable estimation of sample entropy, the length of time series data should be in the range of 10m to 20m, where m is the embedding dimension. The variance of entropy estimates may increase for short duration time series data [8, 10], which may lead to imprecise entropy estimates and increase the probability of inducing undefined entropy values. The finding demonstrated that MNCSE can be a reliable complexity measure in situations where it is not possible to record long duration time series data.

The physiological systems function across multiple temporal scales [35] and like other traditional entropy estimates [6–7, 11] NCSE is single scale based, which provides dynamically incorrect information, i.e., assigned a higher complexity to CHF subject compared to NSR. At scale 2 and above, MNCSE values were higher for NSR subjects than CHF subjects, reflecting the fact that dynamics of NSR subjects are more complex than CHF subjects. On comparing NSR young and elderly subjects, MNCSE values were smaller for elderly subjects than NSR subjects, revealing that complexity decreases with aging. Thus our study suggests the hypothesis that loss of complexity is a generic feature of aging and disease, which is in line with the previous studies [8–10] using MSE analysis. Besides providing dynamically correct information at a majority of temporal scales, MNCSE was more robust in distinguishing NSR and CHF as well as NSR young and NSR elderly subjects.

The variance of MNSCE values were smaller compared to the variance of MSE values within each group, which verifies that MNCSE is statistically a more reliable estimate compared to MSE. In MSE [8–10], the entropy is estimated using sample entropy, whose computation depends on the length of data points in the time and similarity criterion ‘r’ for reliable estimation of complexity. The similarity criterion ‘r’ is expressed as a percentage of standard deviation of the time series (usually 15%) [8, 10]. Thus in the presence of dynamical or observational noise, MSE may provide incorrect information about the dynamics of the system under consideration. On the other hand, the computation of MNCSE depends on the distribution of patterns of sequences of symbols obtained by transforming original time series into symbol sequences, using a specific quantization level and word length. Due to the dependence on the distribution of patterns, the MNCSE is robust in handling the dynamical and observational noise. The analysis of predictive models built on the basis of features extracted through MNCSE and MSE from interbeat interval time series and by using learning algorithms along-with LOOCV and 10 by 10 FCV shows that status of individuals (as healthy and diseased) can be predicted. The presented results also depict that features extracted using MNCSE are more reliable for building more accurate predictive model as compared to features extracted from MSE technique. The results further yielded that MNCSE is robust in case of dynamical and observational noise.

## Conclusion

The biological signals are the outcome of the integrated structural sub-systems and coupling function between them, which operates across multiple spatial and temporal scales. Thus the complexity of these systems is multiscaled, and objective measures that can quantify the complexity at multiple temporal scales are of interest to translational research. Recently, multiscale entropy was proposed to quantify the dynamics physiological system at multiple time scale. In this study, we have proposed a variant of MSE, multiscale normalized corrected Shannon Entropy (MNCSE). The behavior of MNCSE was illustrated using two simulated noise signals (WGN and 1/f noise) and interbeat interval time series data of healthy and pathological subjects. The finding indicated that MNCSE has better performance to illustrate the concept of complexity and in handling the issue of inducing undefined entropy estimates, which is one of the major drawbacks of MSE. The findings indicated that MNSCE is robust in distinguishing cardiac dynamics of healthy, pathological and elderly subjects compared to NCSE, which is the traditional symbolic entropy measure at temporal scale 1. The MNSCE provides better classification in distinguishing the healthy and under-stressed physiological systems compared to MSE. The preliminary results demonstrate that MNCSE can be a valuable tool for quantifying the intrinsic complex nature of physiological systems and for understanding the internal evolution of the system dynamics, when perturbed due to aging or disease. Classification results also reveal that MNCSE is more effective in extracting information from interbeat interval time series than MSE which further strengthens the hypothesis of using MNCSE than MSE.
